# Healthy Food on Instagram Social Network: Vegan, Homemade and Clean Eating

**DOI:** 10.3390/nu13061991

**Published:** 2021-06-09

**Authors:** Ladislav Pilař, Lucie Kvasničková Stanislavská, Roman Kvasnička, Richard Hartman, Ivana Tichá

**Affiliations:** 1 Department of Management, Faculty of Economics and Management, Czech University of Life Sciences Prague, 165 21 Prague, Czech Republic; kvasnickova@pef.czu.cz (L.K.S.); hartman@af.czu.cz (R.H.); ticha@pef.czu.cz (I.T.); 2 Department of Systems Engineering, Faculty of Economics and Management, Czech University of Life Sciences Prague, 165 21 Prague, Czech Republic; kvasnicka@pef.czu.cz

**Keywords:** healthy food, vegan, homemade food, clean eating, social media analysis, instagram

## Abstract

Social media platforms have become part of many people’s lives. Users are spending more and more time on these platforms, creating an active and passive digital footprint through their interaction. This footprint has high research potential in many research areas because understanding people’s communication on social media is essential in understanding their values, attitudes, experiences and behaviors. Researchers found that the use of social networking sites impacts adolescents’ eating behavior. If we define adolescents as individuals between ages 10 and 24 (WHO’s definition), 76% of USA young people at age 18–⁠24 use Instagram, so the Instagram social network analysis is important for understanding young people’s expressions in the context of healthy food. This study aims to identify the main topic associated with healthy food on the Instagram social network via hashtag and community analysis based on 2,045,653 messages created by 427,936 individual users. The results show that users most associate Healthy food with healthy lifestyle, fitness, weight loss and diet. In terms of food, these are foods that are Vegan, Homemade, Clean and Plant-based. Given that young people change their behavior in relation to people’s behavior on social networks, it is possible to use this data to predict their future association with healthy food characteristics.

## 1. Introduction

Social media use is an inherent element of the lives of many people’s lives, particularly for adolescents [1,2,3]. On average, adolescents spend 3.2 h per day on social media [4]. Social networks have become part of their lives, mainly thanks to the ability to create and share their creativity [5], experience and opinion [6], personal attitudes [7] and value [8].

Social media have a significant influence on adolescents in many areas of everyday life. This is a highly important and hot topic, which has been dealt with by many studies, for example: In fashion [9], beauty [10], mental health [11,12,13,14], health behaviors [15], alcohol consumption [16], sexual behavior [17], young people’s health and well-being [15,18], as well as the influence of social media on eating behavior and lifestyle [19]. 

Understanding the factors that influence food selection is fundamental to support the successful translation of dietary goals into consumer behavior [20,21]. Since the social network Instagram is used by 76% of USA young people at age between 18 and ⁠24 [22], social media is an important source of information for understanding consumer behavior related to food behavior [23] through social media analysis, which identifies experience, values and attitudes [24,25,26,27] that users express through social networks. This information can be used both in the area of strategic management and business marketing [7,28] and healthy policy [8].

Thus, providing another area of information that is important for understanding the complex issues of adolescent diet.

This study aims to identify the main topic associated with healthy food on the Instagram social network based on hashtag and community analysis.

The study further aims to answer the following research questions:

What areas do Instagram social network users emphasize through hashtags in the area of healthy food?

What communities exist on the Instagram social network in the area of healthy food?

### 1.1. Theoretical Background

Social media, such as Instagram, changed the way many people consume food [29]. Photos of food are used on Instagram in photographic exchanges to identify and interact with the community [29] and are the second most popular Instagram topic after selfies [30]. Popular social media users called influencers have a strong impact on their followers’ decision-making [10,31,32,33]. In the field of healthy food, they replace the already established food personalities and celebrity chefs and become the creators of healthy eating rules [34] and informal sources of health education [35]. Influencers increasingly change the behavior of individuals in connection with food choice and diet and thus play a crucial role in public health [36].

Because social networks have become an integral part of the lives of many people, as well as food, which is an integral part of everyday life, mauch research is directed towards this area, which also focuses on the influence of social media on the eating habits of adolescents. Research Serenko et al. [3] found the negative influence of daily use of social media by adolescents on their eating habits, namely the habits of eating unhealthy foods. Unhealthy eating is also influenced by the negative influence of social media on the perception of their own body and self in young women [37], because they often support the unhealthy ideal of a very slim body [15]. On the other hand, social media expands the selection of healthy foods by creating an approach to various recipes [38,39] or provide information on diets that can help solve obesity problems [40]. Blundell [19] also claims that social media can turn food preferences into healthier foods.

The exposed area of research is the impact of social media on the health of adolescents, most often focused on mental health [12,41,42]. These studies have shown inconsistent conclusions regarding the influence of social media use on the mental health of adolescents. Some studies draw attention to negative effects, such as the study of Barry et al. [43] that confirmed the correlation between the number of social media accounts and anxiety and depressive symptoms, hyperactivity/impulsivity, anxiety and fear of missing out in adolescents. Nereim et al. [44] draws attention to a stronger combination of passive use of social media with depression than active use (creating contributions) in adolescents. A greater amount of time spent on social media in adolescents is associated with an increased risk of self-harm [45] and depression [41,45,46,47,48,49], whilst lower levels of self-respect [41,45,46] with online harassment [41], bad sleep [3,41,46,50], higher levels of anxiety [46] and poor image of one’s own body [41,51,52]. On the other hand, other studies have not found evidence to confirm the effect of social media on the deterioration of the mental state of adolescents [2,53,54,55]. Social networks cannot be viewed so unilaterally as platforms that only have a negative impact on adolescents. An example is the study Weinstein et al. [56], which identified the positive influence of social media on the mental state of adolescents, such as social connection, social support, affect-enhancing content, shared interests and resources for mental health and coping. Mental health, dietary patterns and many other aspects of adolescent life are highly interrelated areas affected by using social networks in both positive and negative contexts. Due to the fact that online social networks are still a new phenomenon, research of both a quantitative and qualitative character needs to be focused on individual aspects of human life in order to understand these platforms.

#### 1.1.1. Social Media and Social Networks

The terms social networks and social media are often confused with each other. However, there are several differences between them.

#### 1.1.2. Social Media

Authors of The SAGE Handbook of Social Media Research Methods compared various definition of “social media” [57,58,59,60,61]. Individuals, groups, and organizations may use social media to collaborate, communicate, interact, and build community by allowing them to develop, co-create, modify, share, and engage with easily accessible user-generated content [62].

While some definitions stop short of specifying the type of content available, those that do specify content all agree that it is user- or consumer-generated. All definitions indicate what social media does; namely, it allows individuals, communities, and organizations to interact with one another by providing a service that enables them to communicate and collaborate and to create, modify, and share content. The definitions also concur that interactions occur through computer-mediated, web-based services [62]. Social media differ from traditional broadcast media because they directly support or create social networks using information and communication technologies [57]. 

#### 1.1.3. Social Networks

John Barnes introduced the term “Social network” in 1954 [63]. In defining social networks, we start with view by Tichý: A social network is a particular collection of connections between a given group of people, with the added property that the characteristics of these connections as a whole can be used to interpret the social actions of the individuals involved [64]. Social networks portray the relationships that connect us as individuals to our families, communities, organizations, and societies [65]. Individuals, groups, organizations, and related structures form nodes in social networks linked by one or more forms of interdependencies. These interdependencies include, among other things, common beliefs, visions, and ideas; social contacts; kinship; conflict; financial exchanges; trade; joint membership in organizations; and community involvement in events [66]. Furthermore, the social network approach identifies organizations in society as a network of objects (e.g., individuals, groups, organizations) linked by various relationships. Not all pairs of objects are connected explicitly, and several relationships join others. The structure and patterning of these relationships focus on network analysis, defining both their causes and consequences [64]. Social networks are a broad concept that encompasses a wide range of types and functions, with each node having a unique relative value. Nodes are often used to describe events, ideas, or objects [66]. Any series of connections among a group of people and items form a social network. Social network science is a relatively young field, with origins in the early twentieth century pushed forward by scholars including Georg Simmel, Jacob Moreno, or Linton Freeman [67].

Generally, the main differences between online social media and online social network is as follows: social media is defined as a platform where people can share content through not only the social network they created, but also among other users of the platform. To make social media attractive to users, they allow them to create their own social network between people and groups, where they then communicate.

It is a relationship based on friendship or a certain interest.

#### 1.1.4. Social Media Analysis

Social media analysis aims to collect, monitor, analyze, summarize and visualize data from social media platforms, from which valuable patterns and information can be identified [68]. Social Media Analytics is a new interdisciplinary research field that seeks to combine, expand, and adapt approaches for social media data analysis [69]. These basic methods include: Content Analysis, Sentiment Analysis and Opinion Analysis. Content analysis of social media photographs can be used for example for Landscape characterization [70] and Cultural ecosystem service usage [71]. Social media data can help with disaster management [72], detecting traffic accidents [73]. Sentiment analysis in social media analysis allows monitoring social media users opinions about selected products or services or identifying reputations in the context of their competitors and providing them with insight into emerging trends and potential changes in market opinions [74].

For example: Social media sentiment analysis based on COVID-19 [75]. Opinion analysis of social media data can tell us about public’s opinions [76]. What opinions do the users hold? What is the size of each opinion group [77]? 

#### 1.1.5. Social Network Analysis

The study of patterns of relationships between individuals and groups such as organizations and states is the subject of social network analysis, a research technique developed primarily in sociology and communication science. The web can host social networks because it connects people and organizations [78]. The application of network science to the study of human relationships and interactions is known as social network analysis [67].

Social network analysis is a collection of mathematical and analytical methods used to explain the structure and dynamics of natural or artificial networks using network and graph theories [79]. Social network research lies at the crossroads of many disciplines. It’s been linked to sociology, psychology, mathematics, anthropology, and network science for a long time [80]. The actors and their relationships in a particular social context are the two key focuses of social network research, aiming to understand networks and their users. The emphasis on the structure of relationships, ranging from casual acquaintance to close ties, is a distinguishing feature of social network research [66].

Metrics for social networks provide a specific perspective on patterns of knowledge flow and attention giving and receiving. At various levels of research, it provides context for defining key users and their potential impact. Units of analysis for social media network analysis vary and may include a node, a connection, a cluster, or the entire network [81].

Graph density, diameter, reciprocated vertex pair ratio, and a number of linked components are examples of aggregate network metrics that define the network as a whole. They also include network vertex metrics including degree, in-degree, out-degree, betweenness centrality, eigenvector centrality, closeness centrality, PageRank, and clustering coefficient, which can be used to find unique or significant people in a network [82].

Generally, the main differences between social network analysis and social media analysis are: The goal of analyzing social networks is to identify relationships and the strength of these relationships between individual objects in the network. The purpose of the analysis of social media is to analyze the content of communication between these objects.

## 2. Materials and Methods

**Figure 1:**
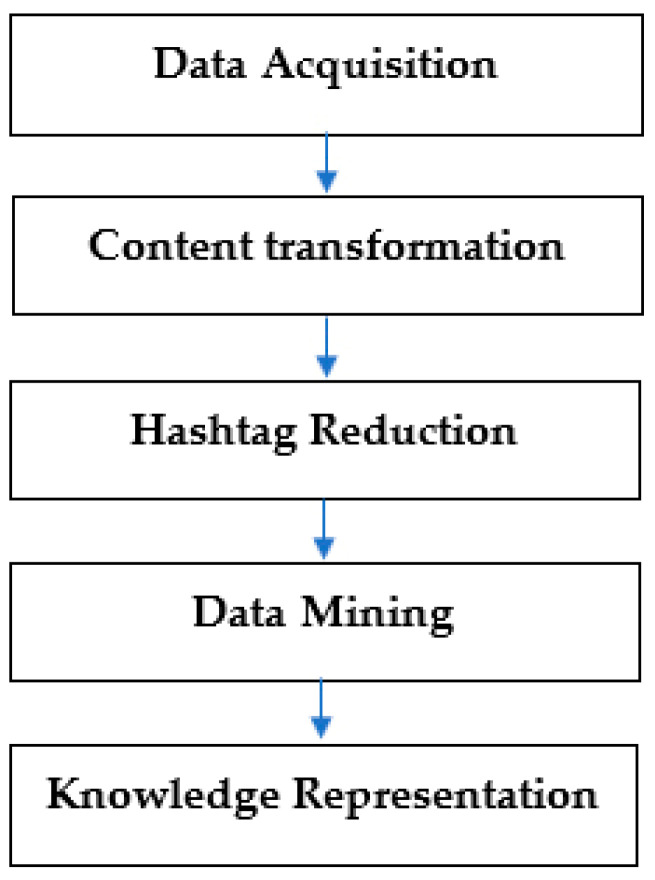
Five phases of social media analysis based on hashtag research (SMAHR) framework process.

**Figure 2:**
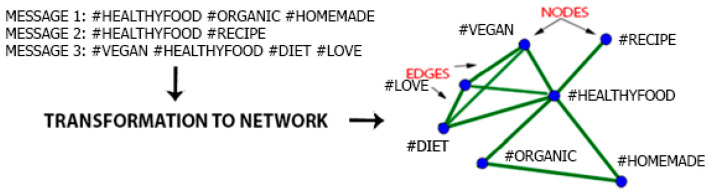
Transformation from the Instagram social network into a hashtag network.

The data analysis was based on the SMAHR framework [83]. SMAHR is a framework that is focused on Social media analysis based on hashtag research. The hashtag is a specific part of the message that begins with a “#” character. On social media, the hashtag has two primary functions: filter posts, where the algorithms of social networks display an archive of messages related to this hashtag (topic) based on a specific hashtag [84], and the second function of hashtags is the way how to emphasize values, experience, attitudes and opinions in the message [24,25,26,27]. In the case of a healthy food, it can emphasize the properties of Gluten-Free, through the hashtag #glutenfree. Gluten-Free is a property of food that may not be obvious from the text and photography. This framework has been already used in research focusing on organic foods [7], farmers’ markets [28], sustainability [85], corporate social responsibility [86] and gamification [87]. The data analysis process based on SMAHR framework consisted of five steps (see Figure 1):

1. **Data acquisition:** Instagram social network was used for data. Instagram Scraper (https://github.com/rarcega/instagram-scraper, accessed on 15 March 2021) was used to obtain data. The software extracted messages that used the hashtag #healthyfood. The extracted data contained 2,045,653 messages created by 427,936 individual users. First, the user ID was encoded by random number algorithm so that it could not be converted back to a user ID. This information was used only to identify the number of users and is in no way associated with the downloaded hashtags. Subsequently, hashtags were extracted from the text of message into a separate database.
2. **Content transformation:** All letters were transformed into lower-case letters to prevent potential duplicates (e.g., the software might consider #Organic, #organic, and #ORGANIC as three different hashtags). The dataset was imported into Gephi 0.9.2 software via the default import module. Hashtag network was created based on hashtag interdependence (see Figure 2). Gephi is a leading visualization and exploration open-source software for graphs and networks [88]. To use social network analysis methods, it was necessary to create a network of hashtags based on the rule: Nodes = Hashtags and Edges = their representation in one message.

For example:

Message: “I love this recipe #healthyfood #homemade #organic” 3 Nodes (#healthyfood, #homemade and #organic) are inserted into the graph and edges are created between these hashtags (because they are all in one message) see Figure 2. If the following message contains the text: “These brownies are amazing, if you want it, here is my #healthyfood #recipe for you”, the hashtag #recipe will be inserted into the graph, which will be connected only with the hashtag #healthyfood, which already in the chart exists from the last message. For the #healthyfood hashtag, the frequency value changes (increase) from 1 to 2.

3. **Hashtag reduction:** Before using the community and modularity analysis, process a hashtag reduction that removes micro-communities. Many micro-communities are caused by an extensive number of hashtags that contain local hashtags, for example, a bakery in the Czech Republic—Prague Motol—#bakerypraguemotol #croissantfrombakerypraguemotol or hashtags created by the users themselves #surnameandname.
4. **Data mining:** The following methods were used to describe the hashtag network:
  (a) *Frequency*: The frequency is a value that expresses the hashtag frequency within a network.
  (b) *Eigenvector centrality*: This is an extension of degree centrality, which measures the influence of hashtags in a network. Eigenvector centrality is calculated based on the premise that connections to hashtags with high values of degree centrality values have a significant influence than links with hashtags of similar or lower values of degree centrality values. A high eigenvector centrality value means that a hashtag is connected to many hashtags with a high degree centrality value. Eigenvector centrality was calculated as follows:
    (1) $$x_{v}=\frac{1}{\lambda }\sum_{t\in M\left(v\right)}\;x_{t}=\frac{1}{\lambda }\sum_{t\in G}\;a_{v,t}x_{t},$$
    where *M*(*v*) denotes a set of adjacent nodes and *λ* is the largest eigenvalue. Eigenvector x can be expressed by Equation (2):
    (2) Ax=λx.
  (c) *Betweenness Centrality*: The value of Betweenness Centrality is highest for a hashtag if the paths between any two hashtags in the network always pass through this hashtag. Hashtags with a high degree of Betweenness Centrality can be referred to as network bottlenecks [89]. These hashtags are important in the network because they act as interconnectors or otherwise as bridges between remote parts of the network. The value of the Betweenness Centrality for the hashtag v in the graph G = (*V*, *E*) is calculated using the following relation:
    (3) $$C_{B}\left(v\right)=\sum_{s\neq v\neq t\in V}\;\frac{\sigma_{st}\left(v\right)}{\sigma_{st}},$$
  (d) *Community analysis and modularity*: The most complex networks contain hashtags that are mutually interconnected to a more significant extent than they are connected to the rest of the network. Cluster of such hashtags are called communities [90]. Modularity represents an index that identifies the cohesion of communities within a given network [91]. The purpose is to identify hashtags communities that are mutually interconnected to a greater degree than other hashtags. Networks with high modularity show strong links between hashtags inside the community and weaker links between hashtags in other communities [92]. The community analysis then identifies the number of different community in the network based on the modularity detection analysis [93], as follows:
    (4) $$\Delta Q=\left[\frac{\sum_{in}^{}+2k_{i,in}}{2m}-{\left(\frac{\sum_{tot}^{}+k_{i}}{2m}\right)}^{2}\right]-\left[\frac{\sum_{in}^{}}{2m}-{\left(\frac{\sum_{tot}^{}}{2m}\right)}^{2}\right],$$
    where ∑*_in_* is the sum of weighted links inside the community, ∑*_tot_* is the sum of weighted links incident to hashtags in the community, *k_i_* is the sum of weighted links incident to hashtag *i*, *k_i_,_in_* is the sum of weighted links going from *i* to hashtags in the community, and *m* is the normalizing factor as the sum of weighted links for the whole graph.
  (e) *Visualization of the network*: Network visualization aims to identify individual communities and their mutual position. After importing the data into the Gephi program, the network’s visualization is concentrated in the basic square without visualizing the different relationships between individual hashtags. This visualization is unsatisfactory in identifying communities and their mutual positions but does not affect the analysis of hashtag-level and network-wide characteristics. In the field of visualization, it is possible to use the ForceAtlas2 algorithm. ForceAtlas2 is an improved version of the ForceAtlas algorithm, which focuses on large networks. This method is based on reduced samples’ visual representation to define network communities and their types [94]. The advantage over ForceAtlas is its speed and ease of computing. The ideal number of hashtags is 10,000–100,000 [95].
5. **Knowledge representation**—a procedure that uses visualization tools to represent the results of data mining. Knowledge representation is based on the synthesis of individual values and outputs from the data evaluation phase.

## 3. Results and Discussion

First, analysis of the occurrence of individual hashtags in relation to Healthy food was used (See Table 1). For the extended version (80 hashtags), see Appendix A.

Based on the analysis of Healthy food through hashtag #healthyfood, it is possible to identify Healthy Lifestyle (through hashtag #healthylifestyle) as the most interconnected area. This is the confirmation of research that confirms the connection of lifestyle and choosing of healthy food [8,32,96]. The 3rd and 4th places can identify two Fitness activities (through hashtag #fitness) and Weight loss (through hashtag #weightloss). This is the link between Healthy food and activities that aim to reduce weight. Based on community analysis (see Table 2), it is the largest community to be extracted in Healthy food.

The most common characteristics of healthy food on Instagram, which were identified by frequency analysis, are vegan (5th place), homemade (6th place), eat clean (17th place) and vegetarian (19th place).

In the 5th place, the vegan area is most communicated (through hashtag #vegan and #veganfood). The vegan area was very often stigmatized and discouraged individuals from a plant-based diet [97,98,99]. The reason was that vegan and vegetarians disrupt social convention related to food [100,101,102]. At present, however, the vegan area is on the rise, both in terms of perception of customers and food producers who have experienced this rise and develop new vegan products, which they also consider one of the sustainability transition paths in the food sector [103]. These results confirm study [104] that showed that vegan and veganism are not a trend followed by a few people in western food culture, but a growing global trend. This also confirms [105], who identified veganism as a megatrend for 2019.

Next (6th) place is occupied by hashtag #homemade, which means Homemade food. In Healthy food, Home food is associated with two basic areas. The first is focused on weight status [106] whilst the second on health balance. Following community analysis (see Table 2), home food on the social network is most widely used as a characteristic of food that is produced at home and popularized by healthy food bloggers. In other words, it is self-presentation on Instagram, where healthy food bloggers present their healthy food. Following the weight analysis, it was possible to characterize the 5 features mostly used in messages containing both hashtags (#healthyfood and #homemade), hashtags #vegan, #vegetarian, #veganfood, #plantbased and #organic). As far as the type of food is concerned, it is mainly salad, then pasta and sweets. For more information, please see Annex 1. Based on the above, there is a strong link between food bloggers who promote homemade food and vegan and vegetarian lifestyle.

Another feature of healthy food on Instagram is clean food (17th place). In the last decade, clean eating has gained popularity as a dietary form (or approach) [107]. Despite this popularity, there is no single definition that defines exactly what the term “clean eating” means. It is generally seen as an approach to food that “promotes the exclusion of processed foods” [108], and is associated with eating “only whole and unprocessed food” [107]. The popularity of clean eating on social media is also confirmed by Ambwani et al. [109], which found that more than 50% of adolescents have knowledge of clean eating from social media or other online sources, and 72% perceive clean eating as a healthy way of eating. A study of [34] points out that significant “digital food Influencers” on social media create and share the meanings of good food which includes clean eating.

The community analysis extrapolated the following four communities: (1) Active Healthy lifestyle (2) Healthy food bloggers (3) Diets (4) Keto (see Table 2).

The largest community was the community focused on “Active Healthy lifestyle”. Active Healthy lifestyle community contained hashtags that were associated with areas such as healthy lifestyle, fitness, fitness motivation, weight loss journey, healthy eating or fit. Health and weight control have become a necessary condition for wellbeing [35] in recent years. Weight reduction is a building block when dealing with obesity [110,111], which is currently a global problem [112,113] and causes many health problems, such as metabolic diseases [114], cancer [115], higher risk of severe COVID-19 [116] or cardiovascular diseases [117]. The topics of this community, however, need not be related only to a healthy solution to obesity. It turns out that health content which promotes weight-management on social media may have unintended consequences, such as recurring weight loss and recovery cycles, chronic stress, avoidance of exercise and depression [35]. As many other studies [37,118,119] have shown, there is a stronger correlation in adolescent girls between the use of social media and the concern about the image of their own body, which may result in eating problems such as pro-eating disorder [120,121,122]. 

The second-largest community was the “Healthy food bloggers” community, in which people shared foods they considered healthy. Healthy food bloggers community contained hashtags that were associated with areas such as foodporn, instafood, instablogger, delicious, etc. The high frequency of these hashtags (specifically delicious, yummy, foodie, foodporn) was also confirmed by the Muralidhara and Paul [123] study, which followed health topics on Instagram. Influencers on Instagram most often share recipes for food and nutritional advice online [36], idealizing healthy food style and creating a community feeling among their followers [29]. They often share not only instructions on how to cook healthy meals, but also how to prepare a perfect and proper lifestyle [34]. Hashtag #foodporn is a phenomenon of recent years when social media users take pictures of meals before or when they are consumed and share them on social networks, especially Instagram [124,125]. The goal is to obtain social consent to shared food through likes, commenting and sharing [126,127,128].

The third community in terms of size is the “Diets” community, containing topics dealing with modern diet approaches such as vegan, vegetarian, gluten free, superfood, dairy free, whole food, etc. As we mentioned in the introduction, Influencer marketing plays a crucial role in public health [36], especially children and adolescents are very easy to influence by popular influencers on social media [33,129]. Popular influencers can motivate adolescents to a healthy diet [32], but many are not qualified nutritionists and their influence on adolescents may lead to health damage [36]. The popularity of ‘free from’ diets on Instagram is confirmed by Goodman and Jaworska [34]. These are often not diets within the meaning of health restrictions such as coeliac disease in gluten free diet, but a free decision for a healthy lifestyle [130,131]. However, the health benefits of these approaches are not often confirmed; on the contrary, there is evidence of possible damage involving possible nutritional deficiencies, financial costs and negative psychosocial consequences as a result of the exclusion of whole food categories [36,132]. With special dietary approaches, the phenomenon of over-focusing on a healthy lifestyle has already been given its professional name—orthorexia nervosa [133,134,135]. Research by Turner and Lefevre [136] revealed that among Instagram users who participated in their study, 49% of them met the criteria for orthoexia. No other social network has had this effect. 

The smallest identified community is the “Keto” community. The community contains themes related to the so-called Ketogenic diets, such as low carb, keto recipes, keto lifestyle, keto weight loss, keto meals, etc. Ketogenic diet is a low-carbohydrate diet with high fat content, which results in the production of ketones by the liver and their uptake as an alternative energy source by the brain [137,138,139]. Although this dietary approach was previously used mainly to treat intractable epilepsy [140], it is currently being promoted as a strategy to combat obesity [141].

### 3.1. Visual Analysis

**Figure 3:**
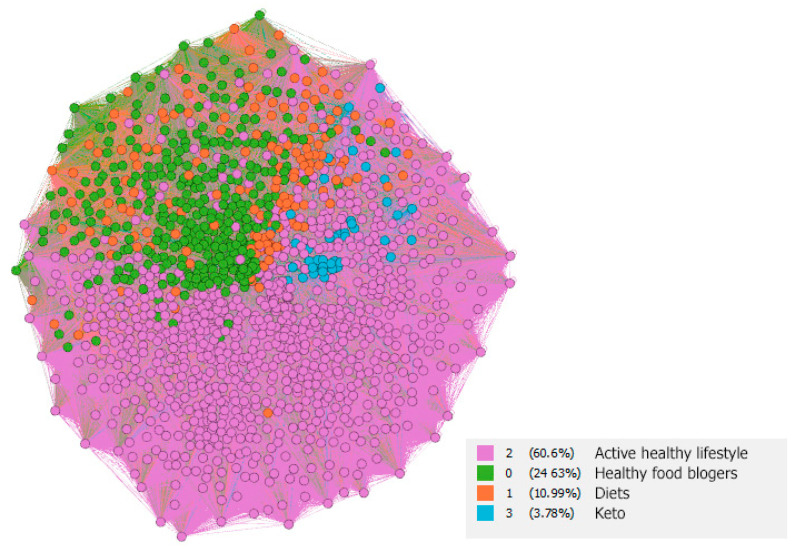
Community visualization in the area of healthy food on the Instagram social network.

Based on a visual analysis where the low polarity of individual communities can be seen, which is confirmed by modularity (0.265), it is possible to identify that the “Keto community” is in the middle of the “Active healthy lifestyle” community, while the “Diets community” is most in the area of “Healthy food bloggers” see Figure 3. 

**Figure 4:**
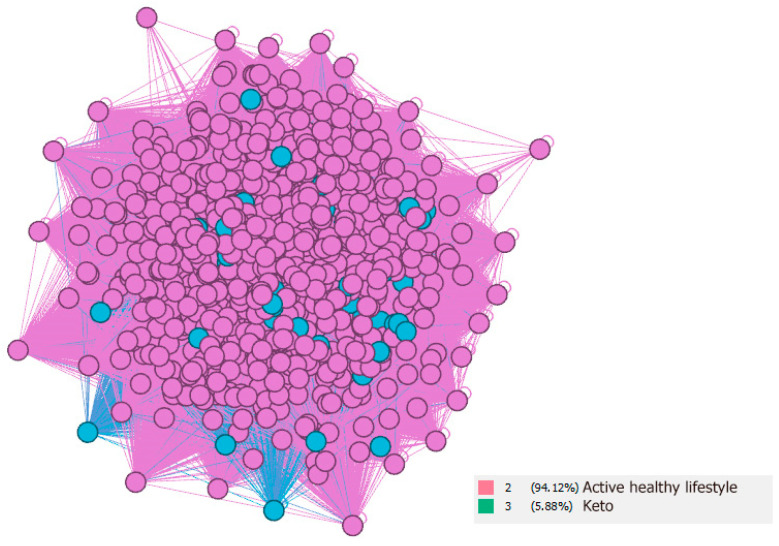
Active healthy lifestyle and Keto community visualization on the Instagram social network in the area of healthy food.

These results can be used mainly from the point of view of strategic marketing, where targeting the area of users who use the Keto diet is primarily in the field of active healthy lifestyle. This is confirmed by the value of betweenness centrality, which can be identified by community bridges (see Table 3), where after expected hashtags (#healthy, #healthyfood and #healthy), the 4th place is taken by #gym, 7th place by #bodybuilding, 8th place by #weightloss and 10th place by #keto. These are the 4 hashtags working as an indicator of active healthy lifestyle, as a bridge between these communities. At the same time, it is possible to identify a very tight relationship between these communities (see Figure 4), where analysis is performed only for hashtags from the “Keto” and “Active healthy lifestyle” communities.

**Figure 5:**
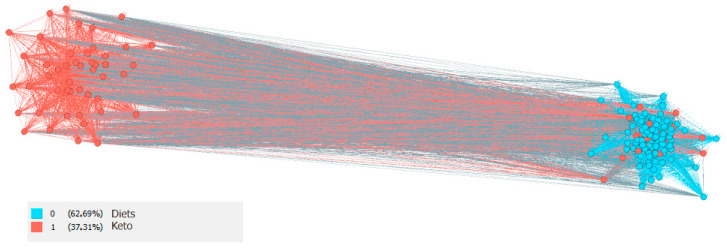
Keto and Diets community visualization on the Instagram social network in the area of healthy food.

When analyzing the two minority communities “Diets” and “Keto”, as indicated in Figure 5 and Table 3. it can be determined that this is a more polarized community where the “Diets” community does not interfere with the “Keto” community, but the “Keto” area is more associated with the “Diets” area. By identifying bridges through betweenness centrality, #glutenfree can be identified as a bridge between these communities.

### 3.2. Limitation of Research

Due to the growing trends in using social media platforms, a social media analysis has a high research potential, however, as another research method, some limitation of this study deserves attention. 

First of all, this research was based on SMAHR (Social Media Analysis based on Hashtag Research) framework [83]. This framework specializes in the use of hashtags for social media analysis. Hashtags are a specific part of message communication where the user emphasizes experiences, personal attitudes or values. It is a method that focuses on areas that users specifically emphasize. The limit is that the analysis does not include messages that the topic contains, but the user does not use hashtags. According to the authors of the SMAHR framework [83], it is a competitive framework to other frameworks that focus on text or object identification in the attached image because it can identify areas that users do not express in the plain text of message or through the attached image.

Second, like other research based on social media analyses, this study focuses only on one social network [7,23,28,85,87,142] and on English hashtags. 

Third, this study did not deal with the geolocation of sending messages. Unlike Twitter, Instagram API does not offer this feature.

Fourth, the study analyzes the current situation and does not deal with predictions for the future. Instagram does not offer the ability to download the date when the message was sent, which is important for predictions.

Sixth, this study does not claim the general perception of healthy food. This study identifies how is healthy food presented on the Instagram social network. For this reason, data includes only the opinions of people who use the Instagram social network, which cannot be applied to the entire population. However, this is essential information in understanding adolescents’ food choices because social networks affect especially young people behavior, and the Instagram social network is used by 76% of USA young people at age 18–24.

### 3.3. Future Research

The results of this study opened many important questions for further research through both quantitative and qualitative methods.

The present research identified values, experience, attitudes and characteristics that Instagram users express in the healthy food area through hashtags.

In future research, it will be necessary to focus on the most frequent identified areas: healthy lifestyle, homemade, weight loss, vegan, clean eating, vegetarian, gluten-free and low carb (keto).

According to experts, some dietary approaches associated on Instagram with healthy food are not unequivocally evaluated as health-friendly (for example, the keto diet [143]). It will be necessary to verify the impact of the identified areas on human health in the future.

A significantly identified hashtag associated with healthy food is the hashtag #weightloss. However, research [120,121,122] has confirmed a stronger correlation between social media use and pro-eating disorder in adolescent girls. The following research would be useful to find out the content of posts with the hashtag #weightloss and their impact on health.

## 4. Conclusions

Based on an analysis of the Instagram social network, it is possible to say that users most associate healthy food with healthy lifestyle. From the point of view of hashtags that are directly drawn to food, the first three places in view of food characteristics are taken by: #vegan, #homemade and #eatclean. Following the different concepts of this area, where diversity occurs, whether vegan is a diet or lifestyle, it has been observed that people on the Instagram social network express themselves on the concept of vegan as lifestyle characteristics. 

Although individuals following a vegan lifestyle are a minority, research indicates a high link in healthy food communication in terms of both the #vegan and #veganfood hashtags usage frequency and extracting own community. Due to the fact that users self-create these characteristics on social networks, it is possible to believe that stigmatization of this area is declining and is an important trend in healthy living and healthy lifestyle.

Many studies have indicated that social networks affect the behavior of adolescents. Due to the fact that Instagram social network is part of the life of many adolescents (for example, 76% of USA young people at age 18–24 use Instagram), we can predict the tendency to Vegan, Homemade, Clean Eat, Plant-based, Gluten Free and Keto diet in these adolescents provided that they follow the healthy food topic. 

Following the community analysis, it is possible to identify “Active healthy lifestyle” as the largest community, which confirms that people on the social network Instagram associate healthy food with an active, healthy lifestyle. The second-largest community is the Influencer Marketing community, meaning that the second-largest community seeks to influence other users in the field of food behaviors, confirming that social networks have the potential to influence people’s behaviors in real life. The third community in terms of size is the “Diets” community, containing topics dealing with modern diet approaches such as vegan, vegetarian, gluten free, superfood, dairy free, whole food. The smallest identified community is the “Keto” community. It is currently a trendy diet, which is spreading on social networks. It is important to note here that this diet should not be used without consulting an expert. The identification of “Keto” community confirms the importance of social network analysis, as it is possible to identify a direction that can lead to health problems but is also associated with healthy food.

The results of this study opened many important questions for further research through both quantitative and qualitative methods. 

These results can be used both in terms of practical use in the area of strategic management of the product portfolio and strategic marketing in terms of defining the value proposition of the product and in terms of theoretical benefits, where it is possible to identify the area of Vegan, Homemade and Clean Eating as areas that social media users associated with a healthy lifestyle.

## Figures and Tables

**Table 1:** Hashtags published in connection with the hashtag #healthyfood on Instagram social Network.

No.	Hashtag	Fr	No.	Hashtag	Fr
1	#healthyfood	2,455,746	21	#plantbased *	141,981
2	#healthylifestyle	938,430	22	#healthyrecipes	141,165
3	#fitness	387,684	23	#gym	139,368
4	#weightloss	317,019	24	#workout	135,423
5	#vegan *	306,533	25	#slimmingworld	132,084
6	#homemade *	304,428	26	#glutenfree *	109,932
7	#diet *	253,830	27	#keto	99,309
8	#nutrition	247,698	28	#restaurant	98,934
9	#dinner	247,485	29	#fitfam	94,002
10	#healthyliving	240,522	30	#organic	93,807
11	#weightlossjourney	233,076	31	#lowcarb	89,631
12	#lunch	232,473	32	#healthylife	83,016
13	#breakfast	220,212	33	#wellness	80,529
14	#cooking	210,120	34	#homecooking	80,334
15	#fit	204,525	35	#slimmingworlduk	77,448
16	#motivation	189,900	36	#bodybuilding	77,145
17	#eatclean *	173,729	37	#weightlosstransformation	74,259
18	#fitnessmotivation	162,123	38	#healthybreakfast	72,708
19	#vegetarian *	144,513	39	#protein	70,788
20	#lifestyle	143,058	40	#chef	70,644

* Linked with food, e.g., # vegan * = # vegan and #veganfood (if both hashtags were in one message, it is counted only once); Fr = Frequency of Hashtag.

**Table 2:** Communities extracted from the reduced network.

Number of Communities *	Size of Community	Name of Community	Key Hashtags
2	60.60%	Active Healthy lifestyle	#healthylifestyle, #healthy, #healthyeating, #fitness, #weightloss, #health, #diet, #weightlossjourney, #fit, #fitnessmotivation
0	24.63%	Healthy food bloggers	#food, #foodie, #foodporn, #instafood, #foodphotograph, y, #yummy, #foodstagram#foodblogger#foodlover#delicious#instagood#homemade#love#dinner#lunch#breakfast#tasty#bhfyp#cooking#foodies#foodgasm#instagram#photooftheday
1	10.99%	Diets	#vegan#vegetarian#veganfood#glutenfree#plantbased#vegetables#veggies#organic#veganrecipes#veggie#veganlife#vegetarianfood#vegetarianrecipes#natural#superfood#green#veganuary#dairyfree#veganfoodshare#vegansofig,#wholefood
3	3.79%	Keto	#keto #lowcarb #ketodiet #easyrecipes #ketorecipes #ketolifestyle #ketomeals #ketofood #ketolife #intermittentfasting #ketogenic #ketoweightloss #ketosis#lchf #sugarfree

* Numbers are linked with Figure 3.

**Table 3:** Betweenes centrality (a) Active healthy lifestyle and Keto Community (b) Diets vs. Keto Communities.

(a) Active Healthy Lifestyle and Keto Community			(b) Diets vs. Keto Communities	
	Hashtag	Betweenes Centrality	Hashtag	Betweenes Centrality
1	#health	0.00201	#veg	0.00692
2	#healthyfood	0.00201	#vegan	0.00374
3	#healthy	0.00177	#veganfood	0.00353
4	#gym	0.00169	#glutenfree	0.00346
5	#healthyeating	0.00156	#easyrecipes	0.00341
6	#healthylifestyle	0.00156	#keto	0.00337
7	#bodybuilding	0.00156	#cauliflower	0.00332
8	#weightloss	0.00152	#veganrecipes	0.00332
9	#keto	0.00152	#lowcarb	0.00331
10	#fitness	0.00149	#goodfood	0.00324

## Data Availability

All data used in this study can be downloaded via the Instagram Scraper (see Data aqusition).

## Appendix A

**Table A1:** Hashtags published in connection with the hashtag #healthyfood on Instagram social Network with non-relevant hashtags.

No	Hashtag	Notes	No	Hashtag	Notes
1	#healthyfood		41	#plantbased	
2	#healthylifestyle		42	#healthyrecipes	
3	#food	*	43	#gym	
4	#healthy	*	44	#workout	
5	#foodie	*	45	#photooftheday	*
6	#foodporn	*	46	#eatclean	
7	#instafood	*	47	#picoftheday	*
8	#healthyeating	*	48	#instadaily	*
9	#foodphotography	*	49	#glutenfree	
10	#fitness		50	#cleaneating	
11	#yummy	*	51	#follow	*
12	#foodstagram	*	52	#keto	
13	#foodblogger	*	53	#restaurant	
14	#foodlover	*	54	#fitfam	
15	#delicious	*	55	#organic	
16	#weightloss		56	#lowcarb	
17	#health	*	57	#eat	*
18	#instagood	*	58	#healthylife	
19	#homemade		59	#like	*
20	#vegan		60	#wellness	
21	#love	*	61	#homecooking	
22	#nutrition		62	#foodiesofinstagram	*
23	#dinner	*	63	#foodpics	*
24	#healthyliving	*	64	#bodybuilding	
25	#diet		65	#weightlosstransformation	
26	#weightlossjourney		66	#exercise	
27	#lunch	*	67	#foodblog	
28	#breakfast	*	68	#healthybreakfast	
29	#tasty	*	69	#protein	
30	#bhfyp	*	70	#chef	
31	#cooking		71	#veganrecipes	
32	#fit		72	#weightlossmotivation	
33	#motivation		73	#salad	
34	#foodies	*	74	#eathealthy	
35	#foodgasm	*	75	#training	
36	#fitnessmotivation		76	#healthychoices	
37	#veganfood		77	#fitfood	
38	#vegetarian		78	#vegetables	
39	#lifestyle		79	#dieta	
40	#instagram	*	80	#yum	

* Remove from Table 1.
